# Hsa_circ_0010235 functions as an oncogenic drive in non-small cell lung cancer by modulating miR-433-3p/TIPRL axis

**DOI:** 10.1186/s12935-021-01764-8

**Published:** 2021-01-25

**Authors:** Furui Zhang, Ruirui Cheng, Ping Li, Chunya Lu, Guojun Zhang

**Affiliations:** Department of Respiratory Medicine, The First Affiliated Hospital of Zhengzhou University, No. 1 Jianshe Road, Zhengzhou, 450052 China

**Keywords:** Hsa_circ_0010235, miR-433-3p, TIPRL, NSCLC, Tumorigenesis

## Abstract

**Background:**

Non-small cell lung cancer (NSCLC) is a threat to human health. Circular RNAs (circRNAs) have been proved to function in NSCLC development. In this study, the role of circRNA hsa_circ_0010235 in NSCLC progression and the possible molecular mechanism were explored.

**Methods:**

Expression of hsa_circ_0010235, miRNA (miR)-433-3p and TOR signaling pathway regulator-like (TIPRL) was examined by quantitative real-time PCR (qRT-PCR). Cell viability and clonogenicity were detected by cell counting kit-8 (CCK-8) assay and colony formation assay, respectively. Flow cytometry was performed to monitor cell apoptosis and cell cycle distribution. Western blot assay was employed to evaluate the protein levels of TIPRL, light chain 3 (LC3)-II/I and p62. Cell metastasis was assessed by Transwell and wound healing assays. The targeted relationship between miR-433-3p and hsa_circ_0010235 or TIPRL was confirmed by dual-luciferase reporter and RNA immunoprecipitation (RIP) assays. Furthermore, the role of hsa_circ_0010235 in vivo was investigated by xenograft assay.

**Results:**

Hsa_circ_0010235 and TIPRL were highly expressed in NSCLC tissues and cells, while miR-433-3p was downregulated. Depletion of hsa_circ_0010235 or gain of miR-433-3p repressed proliferation and autophagy but promoted apoptosis in NSCLC cells. Hsa_circ_0010235 sponged miR-433-3p to upregulate TIPRL expression, so as to affect NSCLC development. Hsa_circ_0010235 knockdown also blocked tumor growth in vivo.

**Conclusion:**

Hsa_circ_0010235 knockdown suppressed NSCLC progression by regulating miR-433-3p/TIPRL axis, affording a novel mechanism of NSCLC progression.

## Background

Lung cancer is one of the most commonly diagnosed malignancies all over the world, with high mortality [1]. Non-small cell lung cancer (NSCLC) is the main subtype of lung cancers, composing ~ 85% of all lung tumors [2]. Due to lacking symptoms at early stages in most NSCLC patients, as well as the high rates of metastasis and relapse, the 5-year survival rate of NSCLC patients is low [3]. Therefore, it’s necessary to explore the involving mechanism of NSCLC pathogenesis in order to develop more effective therapeutic strategies.

Circular RNAs (circRNAs) are a class of non-coding RNA molecules, characterized by the special closed-loop structure, making them resistant to RNase R (an exoribonuclease) [4]. Certain circRNAs have been demonstrated to exert roles in human cancers, serving as cancer-promoting or cancer-suppressing factors [5, 6]. Many circRNAs were reported to affect NSCLC progression, like circSLC25A16, hsa_circ_0043265, circRNA_001010 and hsa_circ_0072309 [7–10]. Back-spliced from aldehyde dehydrogenase 4 family member A1 (ALDH4A1), circRNA hsa_circ_0010235 (Position: chr1:19201875–19216599) was detected to be upregulated in NSCLC tissues [11], while its explicit role in NSCLC development remains to be studied.

MicroRNAs (miRNAs) are also non-coding RNAs, serving as important regulators in tumor development by post-transcriptionally downregulating expression of their target genes, whose length was only about 22 nucleotides [12, 13]. MiRNAs could act as potential diagnosis and therapy biomarkers for NSCLC patients [14]. In addition, miRNAs was implicated in tumorigenesis and progression of NSCLC [15]. MiR-433 was substantiated to be a tumor suppressor in hepatocellular carcinoma (HCC), breast cancer and retinoblastoma [16–18], as well as in NSCLC [19]. In this study, miR-433-3p is predicted to be a target of hsa_circ_0010235 using online tools, while whether the miR-433-3p was involved in hsa_circ_0010235-mediated NSCLC progression needs to be investigated.

Mammalian target of rapamycin (mTOR) signaling was verified to play pivotal roles in tumor cell migration and invasion [20]. TOR signaling pathway regulator-like (TIPRL, the mammalian ortholog of the yeast protein TIP41) was initially recognized in yeast to interact with TAP42 and inhibit mTOR signaling [21]. TIPRL was upregulated in hepatocellular carcinoma (HCC), and it could facilitate TRAIL (a potential anti-cancer agent) resistance of HCC cells [22]. In NSCLC, enforced expression of TIPRL facilitated cell autophagy, and it could serve as an ideal therapeutic target [23]. Here, TIPRL was found to have binding position with miR-433-3p, and the interaction of TIPRL with hsa_circ_0010235-mediated NSCLC progression was also explored.

In this study, the significant upregulation of hsa_circ_0010235 was detected in NSCLC tissues and cells. Functionally, the impact of hsa_circ_0010235 on NSCLC cell proliferation, autophagy, migration, invasion and apoptosis in vitro, as well as on tumor growth in vivo was investigated. The molecular mechanism by which hsa_circ_0010235 affected NSCLC development was also explored.

## Materials and methods

### Clinical tissues and cell lines

Fifty two pairs of NSCLC tissues and corresponding adjacent normal tissues were resected from NSCLC patients enrolled at the First Affiliated Hospital of Zhengzhou University from 2013–2015 and preserved at – 80 ℃. All patients were chosen based on the guidelines supplied by World Health Organization (WHO) and the International Association for the Study of Lung Cancer (IASLC) [24]. Follow-up of these 52 patients was implemented from date of surgery until end of this study or death. All participators signed informed consent.

Human bronchial epithelial cells 16HBE (CL-0249; Procell, Wuhan, China) and NSCLC cells H1299 (ATCC^®^ CRL-5803D; ATCC, Manassas, VA, USA), A549 (ATCC^®^ CCL-185), H1581 (ATCC^®^ CRL-5878) and H23 (ATCC^®^ CRL-5800) were cultured in Dulbecco’s Modified Eagle Medium (Gibco, Grand Island, NY, USA) mixed with 10% fetal bovine serum (Gibco) and 1% penicillin/streptomycin (Gibco) in a humidified incubator at 37 ℃ containing 5% CO_2_.

### Transient transfection

To knock down hsa_circ_0010235 expression in H1299 and A549 cells, small interference RNAs against hsa_circ_0010235 (si-hsa_circ_0010235#1 and si-hsa_circ_0010235#2; KeyGEN Biotech, Nanjing, China) were introduced, with si-NC (KeyGEN Biotech) as negative control. To overexpress hsa_circ_0010235, its overexpression vector (hsa_circ_0010235; GenePharma Co. Ltd., Shanghai, China) was applied, with pCD-ciR (GenePharma Co. Ltd.) empty vector as negative control. MiR-433-3p mimic (miR-433-3p), miR-433-3p inhibitor (anti-miR-433-3p) and their respective negative control (miR-NC and anti-miR-NC) were obtained from GeneCopoeia (Guangzhou, China). Overexpression vector of TIPRL (TIPRL) was constructed by inserting its full-length sequence into pcDNA 3.1 vector (Invitrogen, Carlsbad, CA, USA). Above-mentioned plasmids or oligonucleotides were introduced into NSCLC cells utilizing Lipofectamine 3000 (Invitrogen) following the user’s manual.

### Quantitative real-time PCR (qRT-PCR)

Total RNA was isolated from clinical samples or cells using TRIzol Reagent (Invitrogen). Afterwards, RNA was subjected to reverse transcription for complementary DNA (cDNA) synthesis using M-MLV Reverse Transcriptase (Invitrogen) or miRNA First-Strand Synthesis Kit (Clontech, Mountain View, CA, USA). QRT-PCR assay was performed on ABI Prism7500 Fast Real-Time PCR system (Applied Biosystems, Foster City, CA, USA) with Power SYBR Green Master Mix (Applied Biosystems) or TaqMan miRNA assays (Applied Biosystems). Relative expression was assessed by normalization to Glyceraldehyde-3-phosphate dehydrogenase (GAPDH, for hsa_circ_0010235, ALDH4A1 and TIPRL) or small nuclear RNA U6 (for miR-433-3p and miR-197-3p) using 2^−ΔΔCt^ method [25]. Primers used for qRT-PCR assay were: hsa_circ_0010235, 5′-ACGTCTACCCGGATGACAAG-3′ (sense) and 5′-CTGCGTGAAGGCTAAGACG-3′ (anti-sense); ALDH4A1, 5′-TCTTCCTGAAGGCGGCAGACAT-3′ (sense) and 5′-GCGTCAATCTCCGCTTGGATCA-3′ (anti-sense); miR-433-3p, 5′-GGAGAAGTACGGTGAGCCTGT-3′ (sense) and 5′-GAACACCGAGGAGCCCATCAT-3′ (anti-sense); miR-197-3p, 5′-CACCACCTTCTCCACCCA-3′ (sense) and 5′-GGGACTGGACTTGGAGTC-3′ (anti-sense); TIPRL, 5′-ATGAAGTCGGCGGATGTGGAGA-3′ (sense) and 5′-TTCCAAAGCCAGACCCATGCTG-3′ (anti-sense); GAPDH, 5′-CCACTCCTCCACCTTTGAC-3′ (sense) and 5′-ACCCTGTTGCTGTAGCCA-3′ (anti-sense); U6, 5′-CGCTTCGGCAGCACATATACTAAAAT-3′ (sense) and 5′-CGCTTCACGAATTTGCGTGTCAT-3′ (anti-sense).

### RNase R and actinomycin D treatment

To confirm the stability of hsa_circ_0010235, total RNA extracted from H1299 and A549 cells was digested with RNase R (Geneseed, Guangzhou, China) or not (Mock). 0.5 h later, resulting RNA was subjected to qRT-PCR assay to examine the relative expression of hsa_circ_0010235 and ALDH4A1 mRNA.

Actinomycin D treatment was also applied to validate the stability of hsa_circ_0010235. H1299 and A549 cells were treated with Actinomycin D (an inhibitor of transcription) (Sigma-Aldrich, St. Louis, MO, USA) or dimethyl sulfoxide (DMSO) solution for 0 h, 4 h, 8 h, 12 h or 24 h, followed by qRT-PCR assay to determine the relative expression of hsa_circ_0010235 and ALDH4A1 mRNA.

### Cell counting kit-8 (CCK-8) assay

The current assay was performed to detect cell viability. In brief, H1299 and A549 cells were placed into 96-well plates at 0 h, 24 h, 48 h or 72 h post transfection, then 10 μL CCK-8 reagent was dropped into each well, and incubated for 2 h. Later, optical density (OD) value at 450 nm of each well was recorded using a microplate reader (Bio-Rad Laboratories, Inc., Hercules, CA, USA).

### Colony formation assay

After transfection, 800 H1299 and A549 cells were seeded into 6-well plates. 2 weeks later, generated colonies (mass containing > 50 cells) were fixed using paraformaldehyde, dyed using 0.1% crystal violet solution (Sigma-Aldrich) and photographed, then counted utilizing Image J software (NIH, Bethesda, MD, USA).

### Flow cytometry

Flow cytometry was carried out to monitor cell apoptosis and cell cycle distribution. For cell apoptosis detection, an Annexin V-fluorescein isothiocyanate (FITC)/propidium iodide (PI) Apoptosis Detection Kit (BD Biosciences, Franklin Lakes, NJ, USA) was used. After transfection, H1299 and A549 cells were harvested and re-suspended in binding buffer, then double-stained with 5 μL Annexin V-FITC and 10 μL PI reagent in the dark for 15 min. Subsequently, apoptotic cells (Annexin V-FITC +) were monitored by a flow cytometer (BD Biosciences).

For cell cycle examination, transfected H1299 and A549 cells were immobilized by 75% ethanol and digested with RNase A, followed by the addition of PI solution (Sigma-Aldrich). Later, cells in G0/G1, S and G2/M phases were detected utilizing a flow cytometer.

### Western blot

Clinical samples or cells were lysed in Radio-Immunoprecipitation Assay (RIPA; CWBIO, Beijing, China) with containing protease and phosphatase inhibitors (CWBIO) to isolate protein samples. After quantification using a bicinchoninic acid assay (BCA) protein assay kit (Sigma-Aldrich), 40 μg protein samples were run on 15% sodium dodecyl sulfate (SDS)-polyacrylamide gels (PAGE) and then transferred onto polyvinylidene fluoride (PVDF) membranes (Bio-Rad Laboratories, Inc.). The membranes were subjected to blockage with 5% non-fat milk, incubation with the primary antibody against light chain 3 (LC3) (1:1000; ab192890; Abcam, Shanghai, China), p62 (1:1500; ab109012; Abcam), TIPRL (1:1000; ab71974; Abcam), or loading control GAPDH (1:3000; ab181602; Abcam) at 4 ℃ overnight, and incubation with secondary antibody Goat Anti-Rabbit IgG H&L (HRP) (1:5000; ab205718; Abcam). In the end, immunoreactivity was detected exploiting an enhanced chemiluminescence (ECL) kit and Image J software (Additional file 1).

### Transwell assay

For migration determination, transfected H1299 and A549 cells (1 × 10^5^) suspended in medium without serum were placed onto the top chambers. Complete medium was added to the bottom chambers. After incubation for 48 h, cells migrated through the polycarbonic membrane were fixed using 4% paraformaldehyde and stained with crystal violet, then observed and counted under a microscope (magnification: × 100).

The invasion ability of NSCLC cells was assessed with 5 × 10^5^ cells plated on the top chambers pre-coated with Matrigel (BD Biosciences). Other procedures were same to the migration assay.

### Wound healing assay

After transfection, H1299 and A549 cells were seeded into 24-well plates and maintained until 80–90% confluence. Then a sterile pipette tip (10 μL) was used to scratch the single cell layer across each well to make a scratch wound. The detached cells were washed away using phosphate buffer saline (PBS). Later, serum-free medium was added, followed by capture of phase-contrast images at 0 h or 24 h post incubation exploiting an inverted microscope.

### Targeted prediction, dual-luciferase reporter and RNA immunoprecipitation (RIP) assays

The target miRNAs directly interacted with hsa_circ_0010235 were predicted utilizing the following online tools: circBank (http://www.circbank.cn/), CircInteractome (https://circinteractome.nia.nih.gov/) and starbase (http://starbase.sysu.edu.cn/). Additionally, TargetScan (http://www.targetscan.org/vert_71/) was applied to the forecast the target gene of miR-433-3p.

Dual-luciferase reporter assay (DLRA) was hired to verify the relationship between miR-433-3p and hsa_circ_0010235 or TIPRL 3′ untranslated region (3′UTR). Wild-type (wt-hsa_circ_0010235) and mutant-type (mut-hsa_circ_0010235) luciferase reporter plasmids of hsa_circ_0010235 were designed and constructed by RIBOBIO Co. Ltd. (Guangzhou, China) by inserting the segment of hsa_circ_0010235 containing binding sites with miR-433-3p or mutant ones, respectively. In the same way, luciferase reporter plasmids of TIPRL (wt-TIPRL 3′UTR and mut-TIPRL 3′UTR) were synthesized. Afterwards, generated luciferase reporter plasmid and miR-NC or miR-433-3p were co-transfected into H1299 and A549 cells, followed by luciferase activity measurement using Dual-Luciferase reporter system (Beyotime, Shanghai, China) according to the producer’s instructions. Moreover, Renilla luciferase served as a control luciferase reporter for normalization.

RIP assay was also conducted to testify the relationship between miR-433-3p and hsa_circ_0010235 or TIPRL using the EZ-Magna RIP Kit (Millipore, Billerica, MA, USA) referring to the user’s manual. Briefly, cell lysate of H1299 and A549 cells in lysis buffer was mixed with RIP binding buffer containing magnetic beads conjugated with human Ago2 antibody (1:50; ab32381; Abcam) or IgG antibody (1:50; ab109761; Abcam). Subsequently, RNA was extracted and qRT-PCR assay was implemented to examine the enrichment of hsa_circ_0010235, miR-433-3p and TIPRL.

### In vivo tumorigenesis assay

BALB/c nude mice (male, 5 weeks old) were purchased from Beijing Laboratory Animal Center (Beijing, China). H1299 cells (2 × 10^6^) stably transfected with lentiviral small hairpin RNA (shRNA) targeting hsa_circ_0010235 (sh-hsa_circ_0010235) or sh-NC were subcutaneously inoculated into right flank of nude mice (n = 5). Then, the volume of formed tumors was monitored every 5 days and calculated using the formula: volume (mm^3^) = 0.5 × length × width^2^. 30 days later, all mice were euthanatized, then tumor tissues were removed for weigh, qRT-PCR and Western blot assays.

### Statistical analysis

All data were acquired from 3 independent experiments. Obtained data were analyzed utilizing SPSS 20.0 software (SPSS, Inc., Chicago, IL, USA) and exhibited as mean ± standard deviation. The overall survival rate of NSCLC patients was determined via the Kaplan–Meier method with log-rank test. Correlation among the relative expression levels of hsa_circ_0010235, miR-433-3p and TIPRL in 52 cases of NSCLC tissues was determined by Spearman’s correlation coefficient. Difference significance was calculated by Student’s *t*-test or one-way analysis of variance followed by Tukey’s test, and *P* < 0.05 was recognized to be statistically significant.

## Results

### Hsa_circ_0010235 was highly expressed in NSCLC tissues and cells

**Fig. 1:**
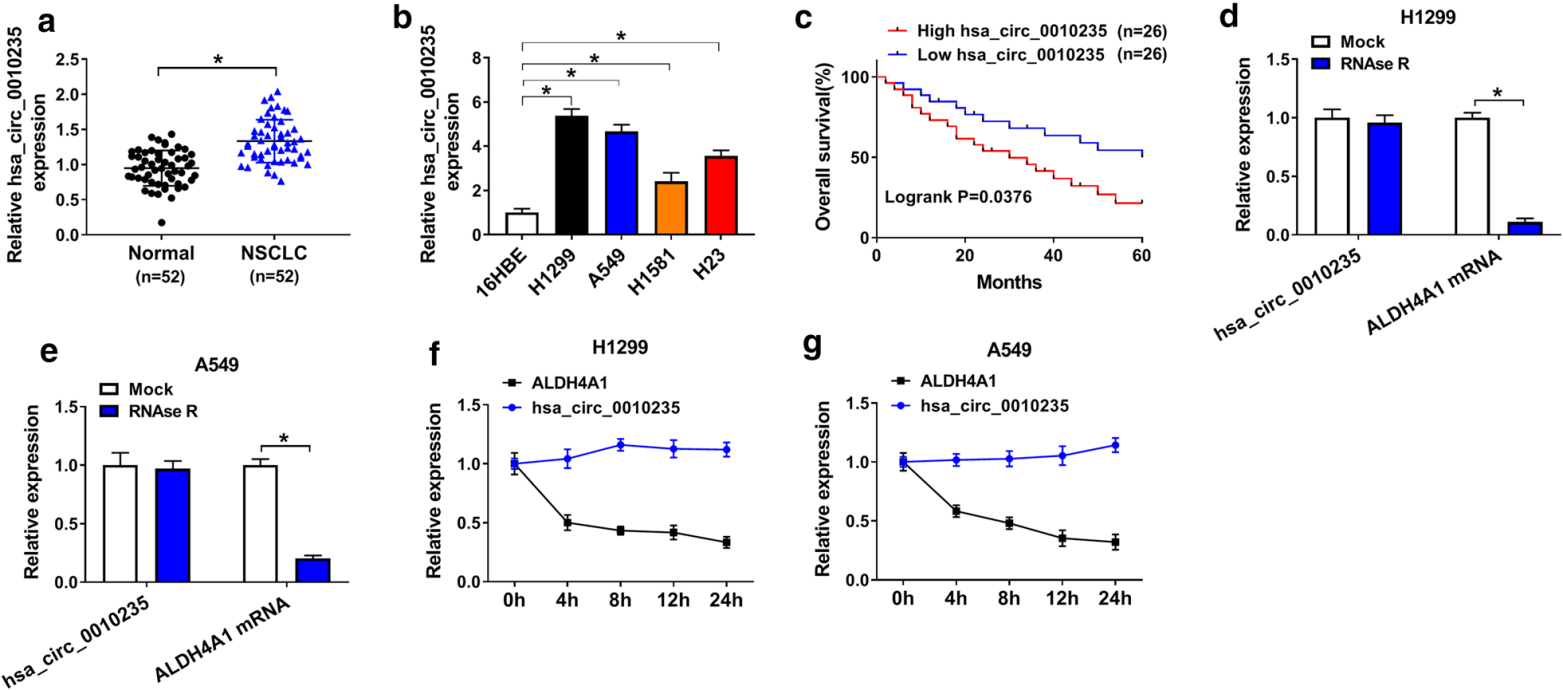
Hsa_circ_0010235 was highly expressed in NSCLC tissues and cells. **a** QRT-PCR assay for the relative expression of hsa_circ_0010235 in NSCLC tissues (n = 52) and normal tissues (n = 52). **b** QRT-PCR assay for the relative expression of hsa_circ_0010235 in 16HBE, H1299, A549, H1581 and H23 cells. **c** Overall survival rate of NSCLC patients with high or low hsa_circ_0010235 expression. **d**, **e** QRT-PCR assay for the relative expression of hsa_circ_0010235 and ALDH4A1 mRNA in RNA isolated from H1299 and A549 cells digested with RNase R or not (Mock). **f**, **g** QRT-PCR assay for the half-life of hsa_circ_0010235 and ALDH4A1 in H1299 and A549 cells treated with Actinomycin D or DMSO. *P < 0.05

At first, the expression level of hsa_circ_0010235 in NSCLC tissues was evaluated by qRT-PCR assay. The data showed that hsa_circ_0010235 expression in NSCLC tissues (n = 52) was higher than that in normal tissues (n = 52) (Fig. 1a). Additionally, hsa_circ_0010235 expression was also increased in H1299, A549, H1581 and H23 cells relative to 16HBE cells (Fig. 1b). Among the four, H1299 and A549 cells were selected for further experiments due to their higher hsa_circ_0010235 expression than H1581 and H23 cells, which have certain representativeness. Follow-up results of these 52 patients revealed that NSCLC patients with higher hsa_circ_0010235 expression had lower overall survival rate. The median expression value of hsa_circ_0010235 was used as the cutoff (*P* = 0.0376) (Fig. 1c). Moreover, clinicopathological characteristics of these 52 NSCLC patients were shown in Table 1, and hsa_circ_0010235 expression was correlated with smoking history, tumor size, TNM stage, lymph node metastasis and recurrence. After digestion with RNase R, ALDH4A1 mRNA expression, rather than hsa_circ_0010235 expression, was obviously reduced in H1299 and A549 cells, suggesting that hsa_circ_0010235 was more stable than ALDH4A1 (Fig. 1d, e). After Actinomycin D disposition, the half-life of hsa_circ_0010235 was more than 24 h, further longer than that of ALDH4A1 mRNA (Fig. 1f, g). Collectively, hsa_circ_0010235 was highly enriched in NSCLC tissues and cells, and high expression of hsa_circ_0010235 could predict low survival rate of NSCLC patients.

**Table 1:** Correlation between the expression of hsa_circ_0010235 in patients with NSCLC and clinicopathological characteristics

Parameters	Case (n = 52)	Hsa_circ_0010235 expression		*P* value
		Low (n = 26)	High (n = 26)	
Gender				
Male	32	15	17	0.569
Female	20	11	9	
Age				
< 60	22	12	10	0.575
≥ 60	30	14	16	
Smoking history				
No	19	13	6	0.044*
Yes	33	13	20	
Pathological type				
Adenocarcinoma	30	16	14	0.364
Squamous cell carcinoma	18	7	11	
Others	4	3	1	
Tumor Size				
< 3 cm	34	24	10	< 0.0001*
≥ 3 cm	18	2	16	
TNM stage				
I	13	10	3	0.025*
≥ II	39	16	23	
Lymph node metastasis				
No	32	22	10	0.0006*
Yes	20	4	16	
Recurrence				
No	27	19	8	0.0023*
Yes	25	7	18	

^*^P < 0.05

### Hsa_circ_0010235 knockdown inhibited proliferation and autophagy but facilitated apoptosis in NSCLC cells

**Fig. 2:**
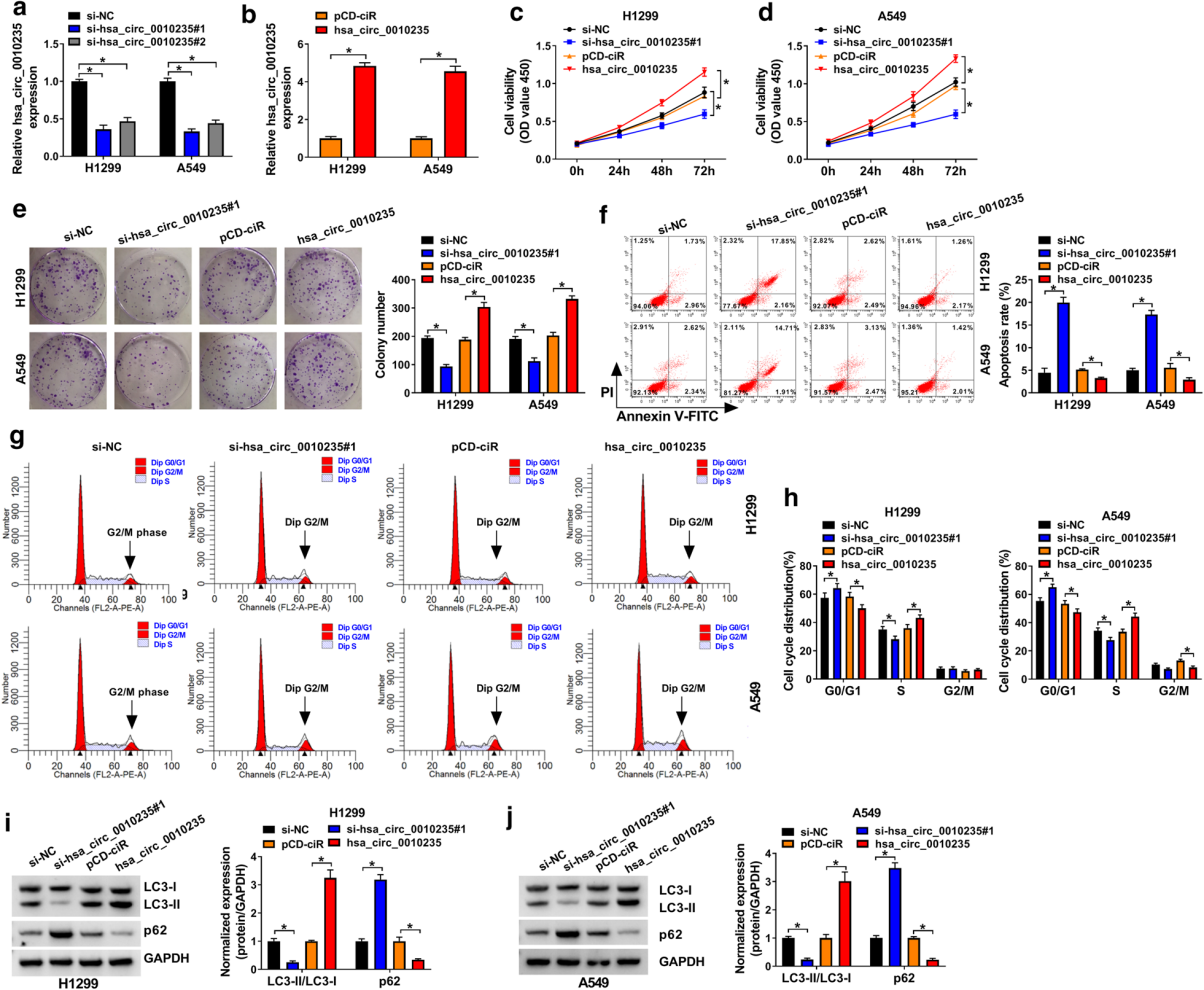
Hsa_circ_0010235 knockdown inhibited proliferation and autophagy but facilitated apoptosis in NSCLC cells. **a** QRT-PCR assay for the relative expression of hsa_circ_0010235 in H1299 and A549 cells transfected with si-NC, si-hsa_circ_0010235#1 or si-hsa_circ_0010235#2. **b** QRT-PCR assay for the relative expression of hsa_circ_0010235 in H1299 and A549 cells transfected with pCD-ciR or hsa_circ_0010235. **c**–**j** H1299 and A549 cells were transfected with si-NC, si-hsa_circ_0010235#1, pCD-ciR or hsa_circ_0010235. **c,** **d** CCK-8 assay for the cell viability of transfected cells. **e** Colony formation assay for the colony formation ability of transfected cells. **f** Flow cytometry for the apoptotic rate of transfected cells. **g,** **h** Flow cytometry for the cell cycle distribution in G0/G1, S and G2/M phases of transfected cells. **i,** **j** Western blot assay for the protein levels of LC3-I, LC3-II and p62 in transfected cells. *P < 0.05

The dysregulation of hsa_circ_0010235 in NSCLC prompted us to investigate the role of hsa_circ_0010235 in NSCLC cells. Si-hsa_circ_0010235#1 and si-hsa_circ_0010235#2 were introduced into H1299 and A549 cells, with si-NC as control. The knockdown efficiency of the two was determined qRT-PCR assay and exhibited in Fig. 2a. Additionally, si-hsa_circ_0010235#1 was chosen for later assays since it induced better knockdown efficiency. And, hsa_circ_0010235 was successfully overexpressed in NSCLC cells via transfection with its overexpression vector, with pCD-ciR as a control (Fig. 2b). CCK-8 assay uncovered that hsa_circ_0010235 knockdown reduced in the cell viability of NSCLC cells; On the contrary, overexpression of hsa_circ_0010235 elevated the cell viability of H1299 and A549 cells (Fig. 2c, d). Moreover, hsa_circ_0010235 knockdown inhibited clonogenicity in NSCLC cells, but overexpression of hsa_circ_0010235 triggered reverse results (Fig. 2e). Data from flow cytometry showed that depletion of hsa_circ_0010235 promoted cell apoptosis and blocked cell cycle at G0/G1 phase, while hsa_circ_0010235 overexpression resulted in opposite results (Fig. 2f–h). Hsa_circ_0010235 knockdown-induced the downregulation of LC3-II and upregulation of p62, as well as the hsa_circ_0010235 overexpression-induced upregulation of LC3-II and downregulation of p62 manifested that hsa_circ_0010235 positively affected autophagy in NSCLC cells (Fig. 2i, j). Above results revealed that hsa_circ_0010235 knockdown inhibited proliferation and autophagy but facilitated apoptosis in NSCLC cells.

### Depletion of hsa_circ_0010235 repressed metastasis of NSCLC cells

**Fig. 3:**
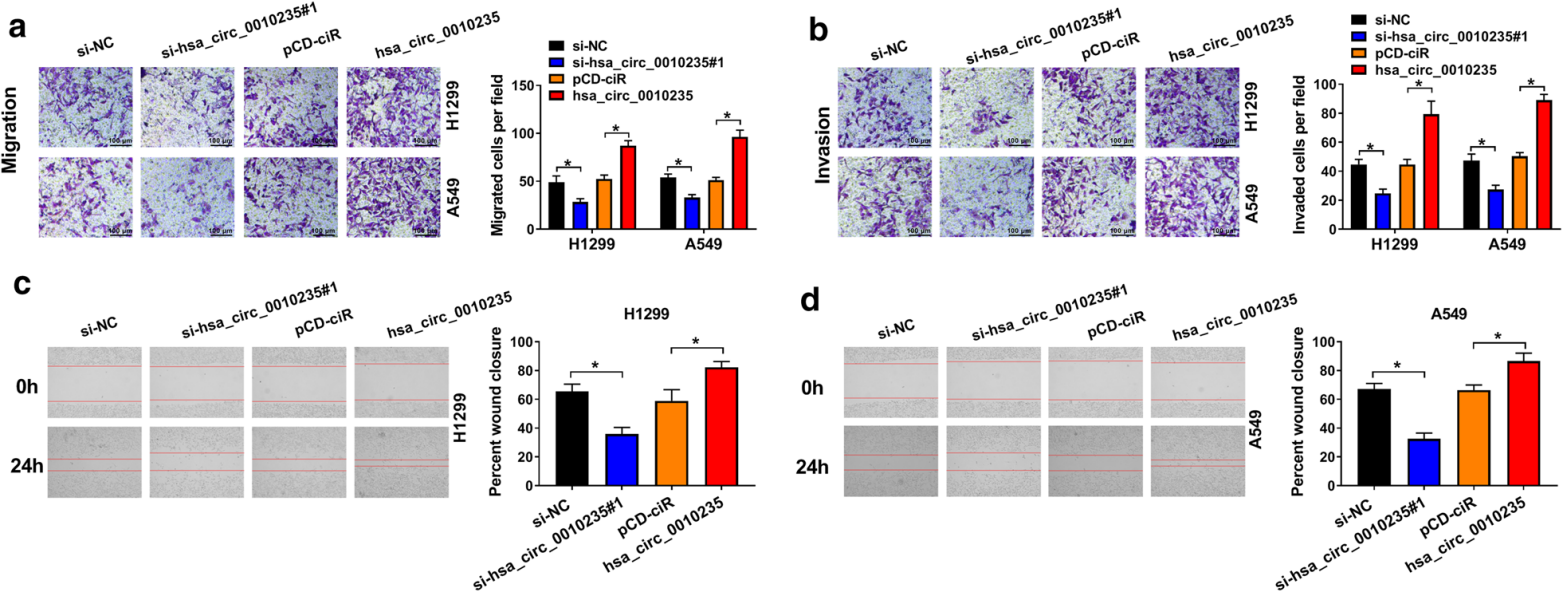
Depletion of hsa_circ_0010235 repressed migration and invasion of NSCLC cells. **a**–**d** H1299 and A549 cells were transfected with si-NC, si-hsa_circ_0010235#1, pCD-ciR or hsa_circ_0010235. **a**, **b** Transwell assay for the migration and invasion of transfected cells. **c**, **d** Wound healing assay for the migration capacity of transfected cells. *P < 0.05. The scale bar indicates 100 μm

The functional effects of hsa_circ_0010235 on migration and invasion of NSCLC cells were also studied. As shown in Fig. 3a, b, hsa_circ_0010235 deficiency efficiently reduced the number of migrated and invaded H1299 and A549 cells; reversely, hsa_circ_0010235 overexpression increased the number of migrated and invaded H1299 and A549 cells. The results of the wound healing assay suggested that silencing of hsa_circ_0010235 apparently inhibited cell motility of H1299 and A549 cells, but upregulation of hsa_circ_0010235 elevated cell motility (Fig. 3c, d). Taken together, depletion of hsa_circ_0010235 repressed metastasis of NSCLC cells.

### Hsa_circ_0010235 served as a sponge for miR-433-3p

**Fig. 4:**
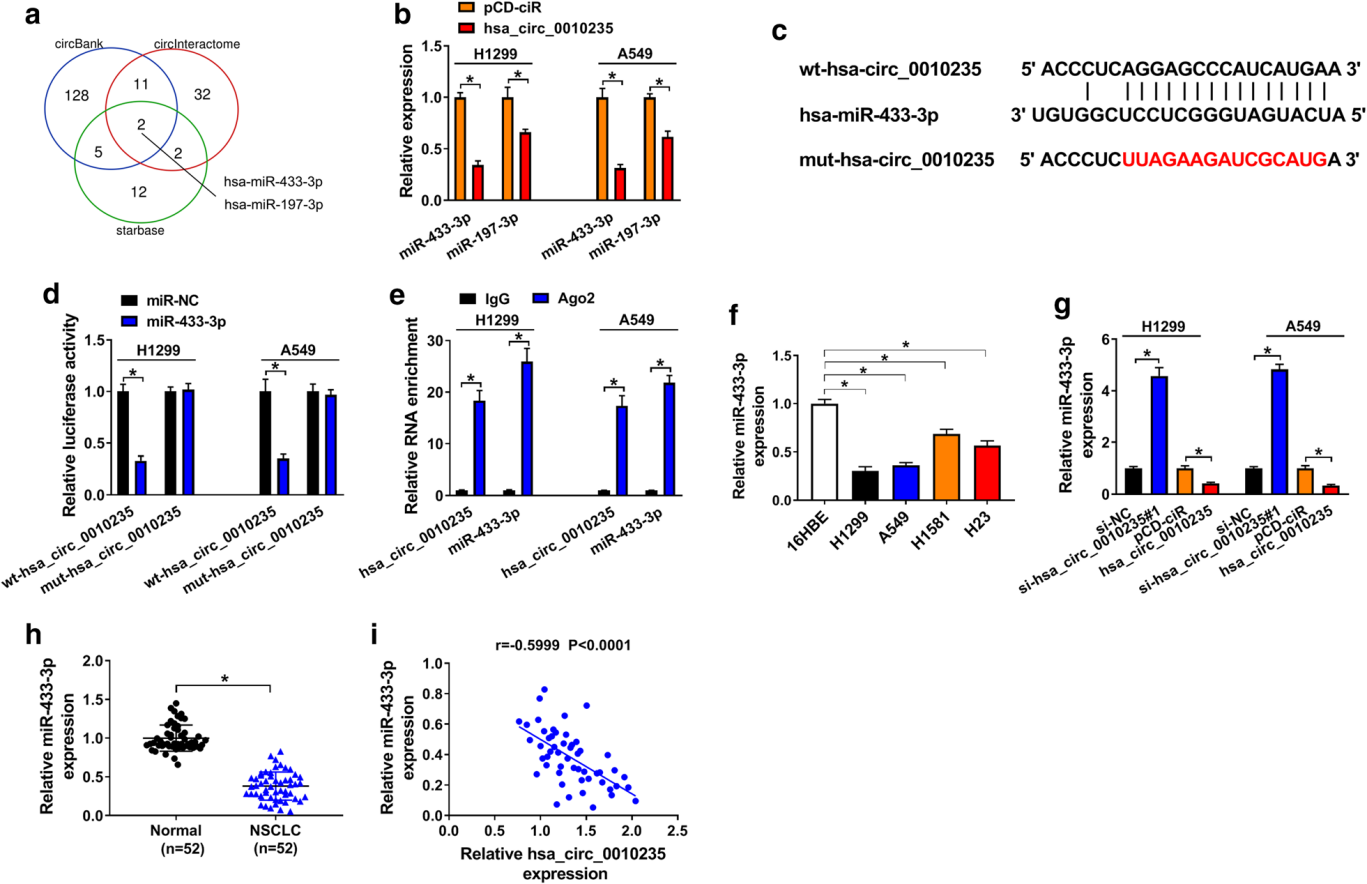
Hsa_circ_0010235 served as a sponge for miR-433-3p. **a** Venn diagram of the overlapping target miRNAs of hsa_circ_0010235 predicted by circBank, CircInteractome and starbase. **b** QRT-PCR assay for the relative expression of miR-433-3p and miR-197-3p in H1299 and A549 cells transfected with pCD-ciR or hsa_circ_0010235. **c** The binding sites between hsa_circ_0010235 and miR-433-3p. **d** DLRA for the luciferase activity of H1299 and A549 cells co-transfected with miR-NC or miR-433-3p and wt-hsa_circ_0010235 or mut-hsa_circ_0010235. **e** RIP assay for the target relationship between hsa_circ_0010235 and miR-433-3p in H1299 and A549 cells. **f** QRT-PCR assay for the relative expression of miR-433-3p in 16HBE, H1299, A549, H1581 and H23 cells. **g** QRT-PCR assay for the relative expression of miR-433-3p in H1299 and A549 cells transfected with si-NC, si-hsa_circ_0010235#1, pCD-ciR or hsa_circ_0010235. **h** QRT-PCR assay for the relative expression of miR-433-3p in NSCLC tissues (n = 52) and normal tissues (n = 52). **i** Spearman’s correlation analysis for the expression levels of hsa_circ_0010235 and miR-433-3p in 52 NSCLC tissues. *P < 0.05

Admittedly, circRNAs could exert their functions by sponging miRNAs [26]. Here, circBank, CircInteractome and starbase were used to search the potential target miRNAs of hsa_circ_0010235. As shown in Fig. 4a, both miR-433-3p and miR-197-3p were found to have binding region with hsa_circ_0010235, forecasted by these three tools. Furthermore, qRT-PCR assay showed that gain of hsa_circ_0010235 reduced the expression of miR-433-3p and miR-197-3p, especially miR-433-3p, so miR-433-3p was selected for later investigation (Fig. 4b). The binding sites between hsa_circ_0010235 and miR-433-3p were exhibited in Fig. 4c. To confirm the relationship between hsa_circ_0010235 and miR-433-3p, DLRA and RIP assay were executed. As shown in Fig. 4d, introduction of miR-433-3p triggered about 60% reduction in luciferase activity of H1299 and A549 cells co-transfected with wt-hsa_circ_0010235, while it had no significant impact on luciferase activity of H1299 and A549 cells co-transfected with mut-hsa_circ_0010235. Additionally, both hsa_circ_0010235 and miR-433-3p were highly enriched in Ago2 pellets relative to IgG pellets (Fig. 4e). We found that miR-433-3p was downregulated in NSCLC cells (Fig. 4f) and NSCLC tissues (Fig. 4h), in contrast to corresponding control. And, miR-433-3p expression in NSCLC tissues was negatively correlated with hsa_circ_0010235 expression (r = − 0.5999, *P* < 0.0001) (Fig. 4i). QRT-PCR assay witnessed the hsa_circ_0010235 knockdown-induced upregulation of miR-433-3p and the hsa_circ_0010235 overexpression-induced downregulation of miR-433-3p (Fig. 4g). Therefore, miR-433-3p was a target of hsa_circ_0010235.

### MiR-433-3p acted as a suppressor in NSCLC development

**Fig. 5:**
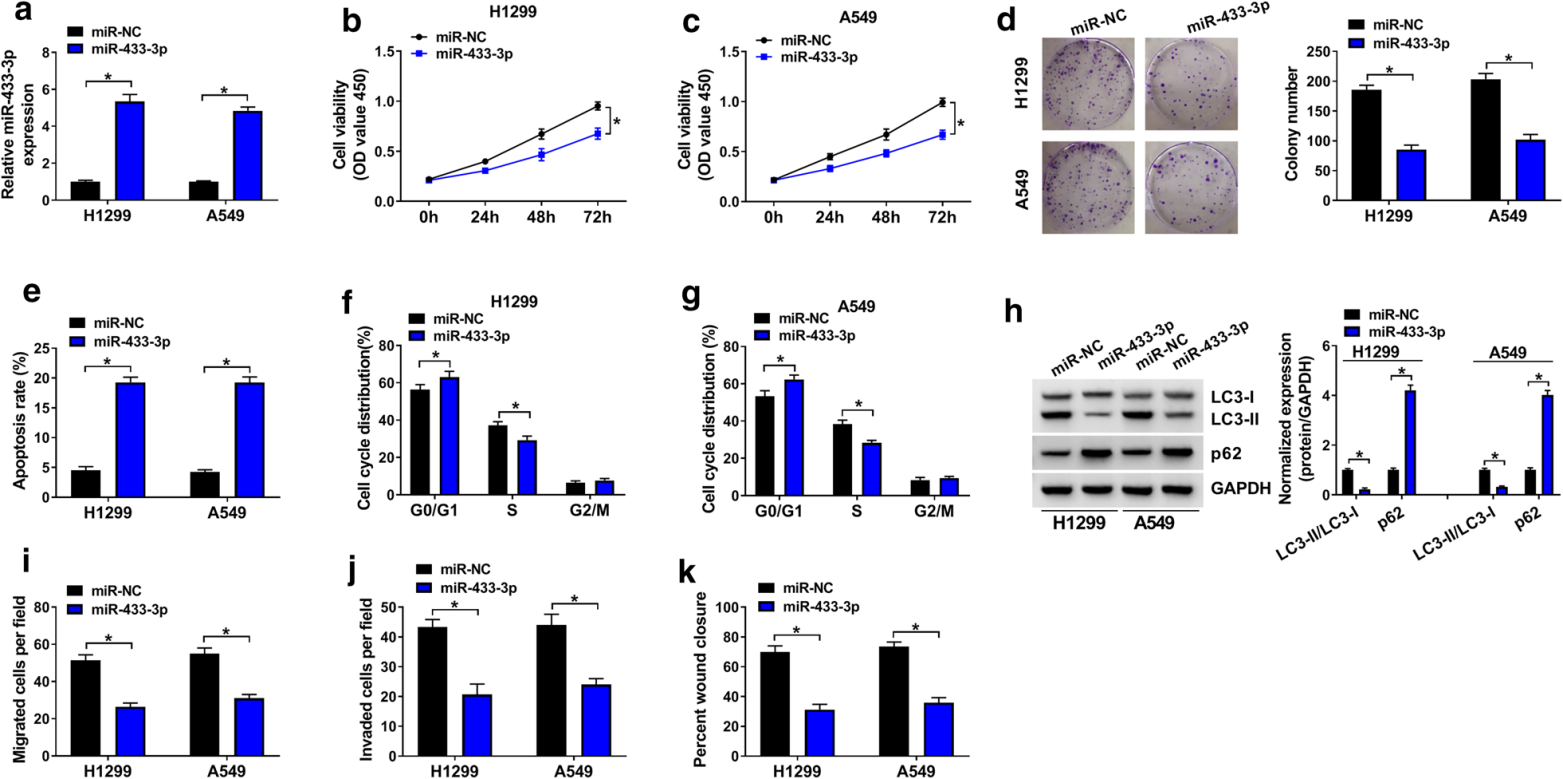
MiR-433-3p acted as a suppressor in NSCLC development. H1299 and A549 cells were transfected with miR-NC or miR-433-3p. **a** QRT-PCR assay for the relative expression of miR-433-3p in transfected cells. **b**, **c** CCK-8 assay for the cell viability of transfected cells. **d** Colony formation assay for the colony formation ability of transfected cells. **e** Flow cytometry for the apoptotic rate of transfected cells. **f**, **g** Flow cytometry for the cell cycle distribution in G0/G1, S and G2/M phases of transfected cells. **h** Western blot assay for the protein levels of LC3-I, LC3-II and p62 in transfected cells. **i**, **j** Transwell assay for the migration and invasion of transfected cells. **k** Wound healing assay for the migration capacity of transfected cells. *P < 0.05

The role of miR-433-3p in NSCLC development was then explored. Firstly, miR-433-3p expression in H1299 and A549 cells was obviously elevated due to the transfection with miR-433-3p, miR-NC serving as a control (Fig. 5a). Following functional assay manifested that gain of miR-433-3p efficiently inhibited cell viability (Fig. 5b, c) and clonogenicity (Fig. 5d), promoted cell apoptosis (Fig. 5e), reduced cell cycle at S phase (Fig. 5f, g), inhibited cell autophagy (Fig. 5h) and metastasis (Fig. 5i–k). To sum up, miR-433-3p acted as a suppressor in NSCLC development.

### TIPRL was a target of miR-433-3p

**Fig. 6:**
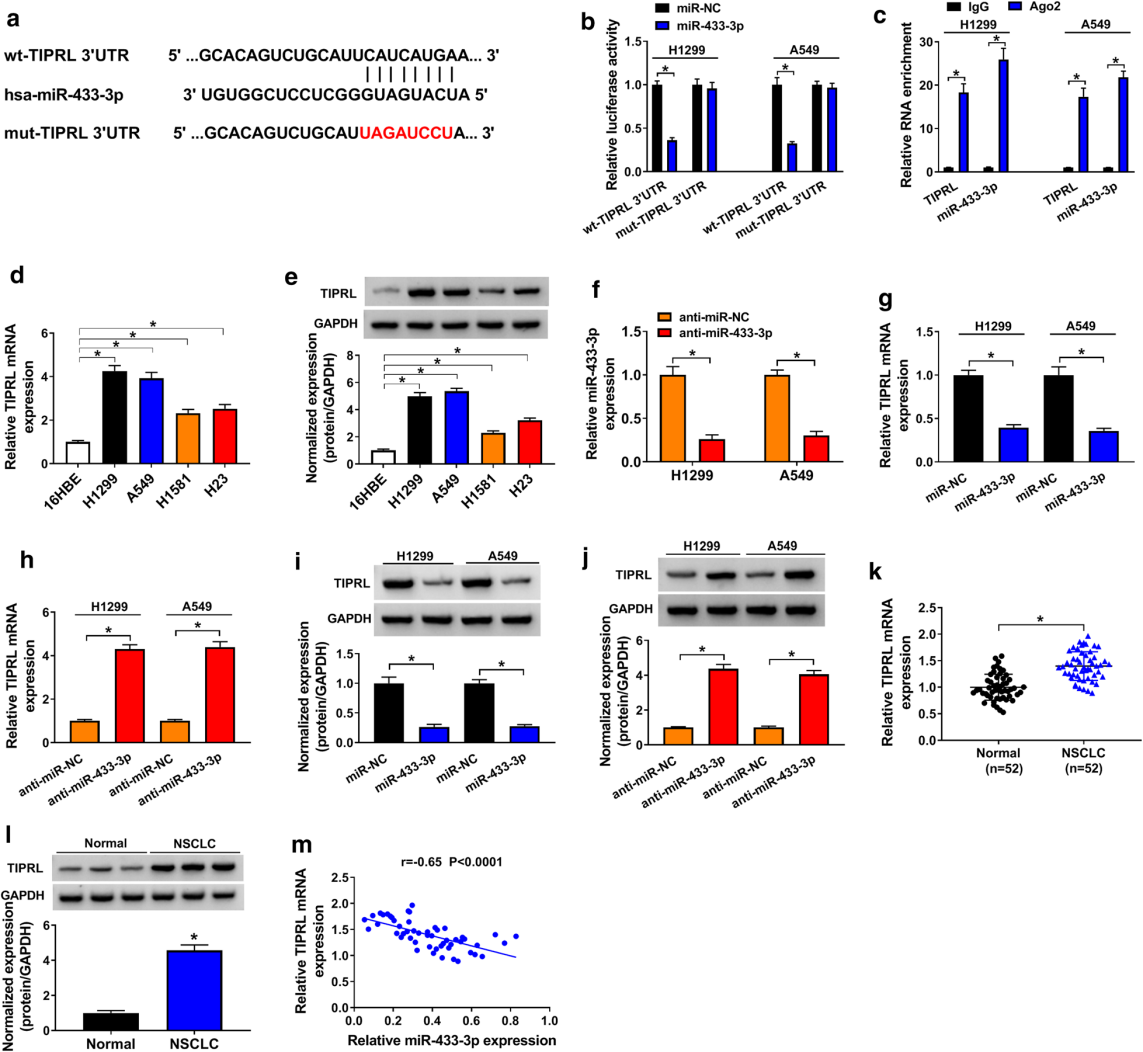
TIPRL was a target of miR-433-3p. **a** The binding sites between miR-433-3p and TIPRL. **b** DLRA for the luciferase activity of H1299 and A549 cells co-transfected with miR-NC or miR-433-3p and wt-TIPRL 3′UTR or mut-TIPRL 3′UTR. **c** RIP assay for the target relationship between miR-433-3p and TIPRL in H1299 and A549 cells. **d**, **e** QRT-PCR and Western blot assays for the mRNA **d** and protein **e** expression levels of TIPRL in 16HBE, H1299, A549, H1581 and H23 cells. **f** QRT-PCR assay for the relative mRNA expression of TIPRL in H1299 and A549 cells transfected with anti-miR-NC or anti-miR-433-3p. **g**, **i** QRT-PCR assay for the relative mRNA **g** and protein **i** expression levels of TIPRL in H1299 and A549 cells transfected with miR-NC or miR-433-3p. **h**, **j** QRT-PCR assay for the relative mRNA **h** and protein **j** expression levels of TIPRL in H1299 and A549 cells transfected with anti-miR-NC or anti-miR-433-3p. **k** QRT-PCR assay for the relative mRNA expression of TIPRL in NSCLC tissues (n = 52) and normal tissues (n = 52). **l** Western blot assay for the protein level of TIPRL in NSCLC tissues and normal tissues. **m** Spearman’s correlation analysis for the expression levels of miR-433-3p and TIPRL mRNA in 52 NSCLC tissues. *P < 0.05

MiRNAs could post-transcriptionally downregulate their target mRNAs, so as to inhibit the function of mRNAs [27]. TargetScan was exploited to predict the target gene of miR-433-3p, and the binding sites between miR-433-3p and TIPRL were shown in Fig. 6a. Data of DLRA indicated that enforced expression of miR-433-3p significantly reduced the luciferase activity of H1299 and A549 cells co-transfected with wt-TIPRL 3′UTR when compared with cells co-transfected with mut-TIPRL 3′UTR (Fig. 6b). Following RIP assay also disclosed the target relationship between miR-433-3p and TIPRL (Fig. 6c). TIPRL expression was upregulated in NSCLC cells (H1299, A549, H1581 and H23) in comparison with 16HBE cells, at both mRNA (Fig. 6d) and protein (Fig. 6e) levels. H1299 and A549 cells with miR-433-3p inhibition were established by transfection with miR-433-3p inhibitor, and cells transfected with anti-miR-NC served as control (Fig. 6f). Additionally, gain of miR-433-3p apparently downregulated the mRNA (Fig. 6g) and protein (Fig. 6i) levels of TIPRL, while miR-433-3p inhibition triggered reverse results (Fig. 6h, j). Moreover, TIPRL mRNA expression was upregulated in NSCLC tissues when compared to normal tissues (Fig. 6k), and was inversely correlated with miR-433-3p in NSCLC tissues (r = − 0.65, *P* < 0.0001) (Fig. 6m). Likewise, the protein level of TIPRL was increased in NSCLC tissues in contrast to normal tissues (Fig. 6l). Above results suggested that miR-433-3p targeted TIPRL and negatively regulated its expression in NSCLC cells.

### Silencing of miR-433-3p or overexpression of TIPRL could attenuate hsa_circ_0010235 knockdown-induced inhibitory effects on NSCLC progression

**Fig. 7:**
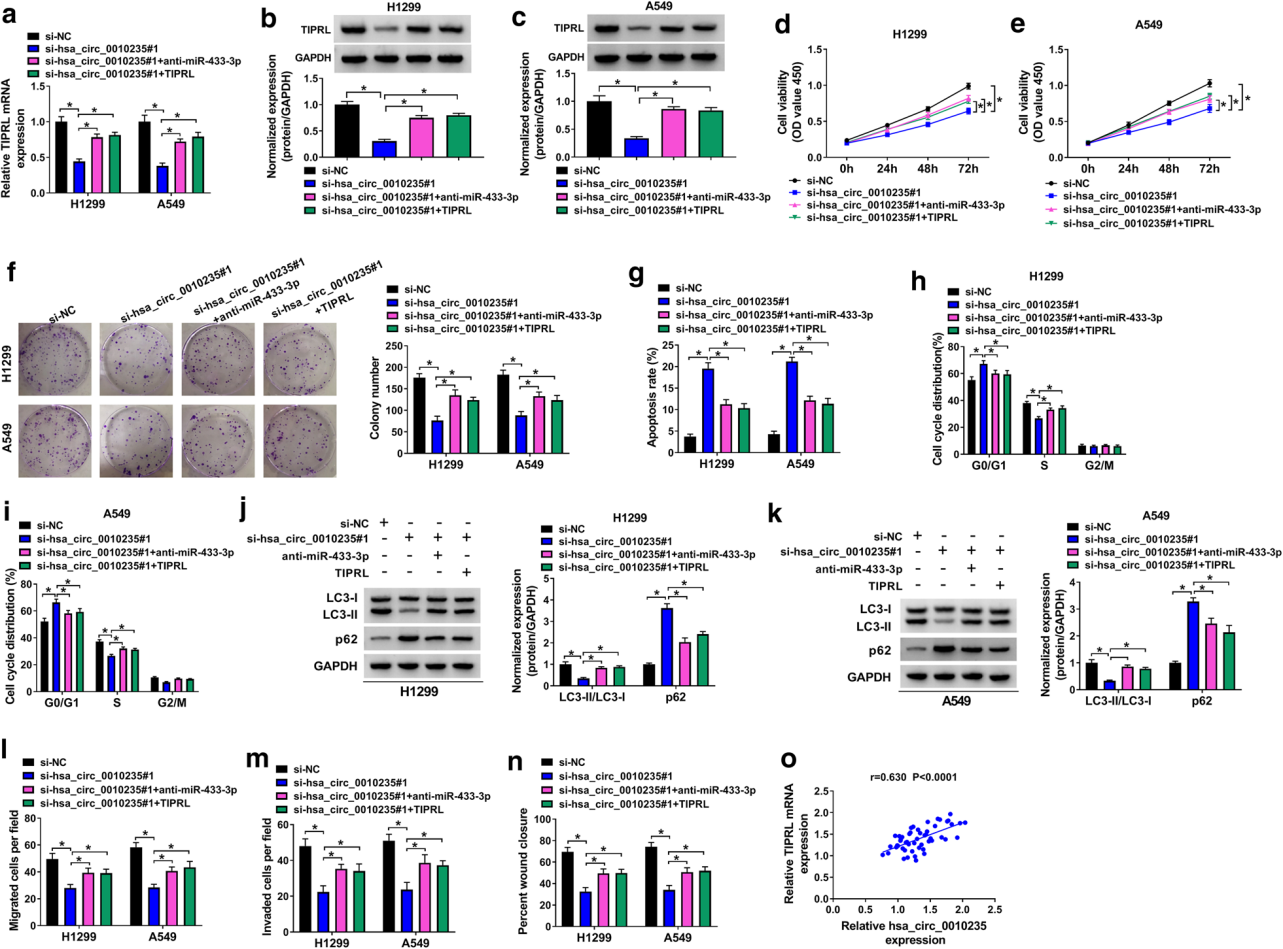
Silencing of miR-433-3p or overexpression of TIPRL could attenuate hsa_circ_0010235 knockdown-induced inhibitory effects on NSCLC progression. H1299 and A549 cells were transfected with si-NC, si-hsa_circ_0010235#1, si-hsa_circ_0010235#1 + anti-miR-433-3p or hsa_circ_0010235#1 + TIPRL. **a** QRT-PCR assay for the relative mRNA expression of TIPRL in transfected cells. **b**, **c** Western blot assay for the protein level of TIPRL in transfected cells. **d**, **e** CCK-8 assay for the cell viability of transfected cells. **f** Colony formation assay for the colony formation ability of transfected cells. **g** Flow cytometry for the apoptotic rate of transfected cells. **h**, **i** Flow cytometry for the cell cycle distribution in G0/G1, S and G2/M phases of transfected cells. **j**, **k** Western blot assay for the protein levels of LC3-I, LC3-II and p62 in transfected cells. **l**, **m** Transwell assay for the migration and invasion of transfected cells. **n** Wound healing assay for the migration capacity of transfected cells. **o** Spearman’s correlation analysis for the expression levels of hsa_circ_0010235 and TIPRL mRNA in 52 NSCLC tissues. *P < 0.05

Having known that hsa_circ_0010235 targeted miR-433-3p and miR-433-3p targeted TIPRL, we further explored the involvement of the hsa_circ_0010235/miR-433-3p/TIPRL axis in NSCLC development. We found that hsa_circ_0010235 knockdown could downregulate TIPRL expression in H1299 and A549 cells, while miR-433-3p inhibitor or overexpression of TIPRL largely relieved it (Fig. 7a–c). In addition, hsa_circ_0010235 knockdown-induced the declined cell viability (Fig. 7d, e) and clonogenicity (Fig. 7f), increased cell apoptosis (Fig. 7g), reduced cell cycle at S phase (Fig. 7h, i), repressed cell autophagy (Fig. 7j, k) and metastasis (Fig. 7l–n) were all attenuated by depletion of miR-433-3p or introduction of TIPRL. Moreover, expression of TIPRL mRNA in NSCLC tissues was positively correlated with that of hsa_circ_0010235 (Fig. 7o). Therefore, hsa_circ_0010235 functioned in NSCLC development via regulating miR-433-3p/TIPRL axis.

### Depletion of hsa_circ_0010235 blocked tumor growth in vivo

**Fig. 8:**
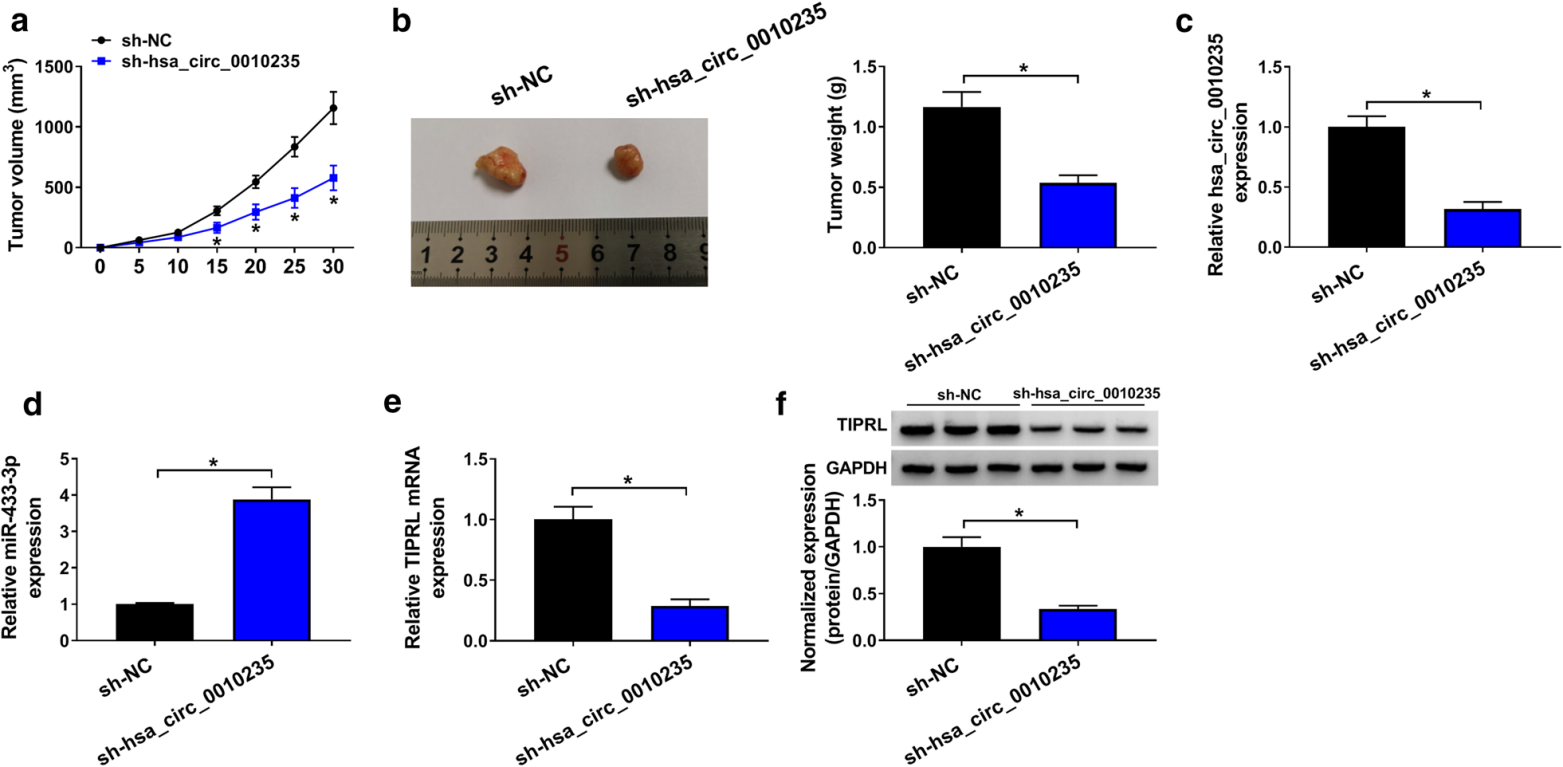
Depletion of hsa_circ_0010235 blocked tumor growth in vivo. Nude mice were injected with H1299 cells stably expressing sh-hsa_circ_0010235 or sh-NC (n = 5). **a** Volume of formed tumors measured every 5 days. **b** Weight of formed tumors measured after 30 days. **c**, **d** QRT-PCR assay for the relative expression of hsa_circ_0010235 **c** and miR-433-3p **d** in formed tumors. **e**, **f** QRT-PCR and Western blot assays for the mRNA **e** and protein **f** levels of TIPRL in formed tumors. *P < 0.05

H1299 cells stably expressing sh-hsa_circ_0010235 or sh-NC were subcutaneously inoculated into nude mice (n = 5) to construct xenograft model in vivo, divided into sh-hsa_circ_0010235 group and sh-NC group. Results showed that both the tumor size (Fig. 8a) and the tumor weight (Fig. 8b) were less in sh-hsa_circ_0010235 group than those of sh-NC group. Moreover, expression of hsa_circ_0010235 (Fig. 8c) and TIPRL (Fig. 8e, f) was downregulated, while miR-433-3p (Fig. 8d) was upregulated in tumor tissues of sh-hsa_circ_0010235 group relative to those of sh-NC group. Taken together, depletion of hsa_circ_0010235 inhibited tumor growth in vivo.

## Discussion

Circular RNAs (circRNAs) with dysregulated expression were associated with growth and metastasis of NSCLC, which could serve as biomarkers of lung cancer diagnosis, prognosis and therapy response [28]. In this project, the functional effects of hsa_circ_0010235 on NSCLC progression were corroborated for the first time. Depletion of hsa_circ_0010235 could repress NSCLC cell proliferation, autophagy, mobility and tumorigenesis, highlighting its carcinogenic role in NSCLC.

Increasing evidence showed that circRNAs were involved in the pathophysiological processes of NSCLC, affecting most cellular behaviors of tumor cells, including proliferation, migration, invasion, cell cycle epithelial-mesenchymal transition and drug resistance [29]. With the development of sequencing technology and computational algorithms, increasing circRNAs were discovered to be meritorious in human cancers [30]. Through human circular RNA microarray and qRT-PCR assay, hsa_circ_0010235 was previously reported to be aberrantly upregulated in NSCLC tissues relative to normal tissues [11]. In this study, we also found the upregulation of hsa_circ_0010235 in NSCLC tissues and cells. Also, our data initially showed the hsa_circ_0010235 knockdown-induced inhibitory effects on cell proliferation, autophagy, metastasis and tumorigenesis of NSCLC cells, suggesting that hsa_circ_0010235 functioned as an oncogene in NSCLC.

With diverse functions, circRNAs are involved in cancer development in different ways, such as acting as sponges or decoys of miRNAs or proteins, scaffolds or transporters [31]. Classically, circRNAs function by sponging miRNAs. In our study, three online tools were utilized to forecast the target miRNAs of hsa_circ_0010235, miR-433-3p was identified as candidates by DLRA and RIP assay. Shi et al. alleged that miR-433-3p could decelerate esophageal squamous cell carcinoma (ESCC) proliferation and metastasis by downregulating growth factor receptor-bound protein 2 (GRB2) [32]. MiR-433-3p also inhibited cell growth, invasion and migration in human glioma by targeting cyclic adenosine monophosphate (AMP) response element-binding protein (CREB) [33]. Other works also highlighted the tumor-suppressor role of miR-433-3p in hepatocellular carcinoma (HCC), breast cancer, retinoblastoma and NSCLC [16–19]. As reported by Li et al. miR-433-3p was downregulated in NSCLC tissues, and its overexpression triggered anti-proliferative and anti-metastatic effects on NSCLC cells [19]. Consistently, we also detected the downregulation of miR-433-3p in NSCLC tissues and cells. Functionally, miR-433-3p repressed NSCLC cell proliferation, autophagy and metastasis. Furthermore, inhibition of miR-433-3p weakened hsa_circ_0010235 knockdown-induced repressed impact on NSCLC progression.

Generally, miRNAs could bind to the 3′UTRs of their target mRNAs to block the translation, so as to affect their functions [34]. In this study, TargetScan predicted that 3′UTR of TIPRL had complementary sites with miR-433-3p, and the target relationship was confirmed by DLRA and RIP assay. Former literature testified that TIPRL deficiency promoted the apoptosis of lung cancer H1299 cells disposed by cisplatin, suggesting its important role in lung cancer [35]. TIPRL expression was increased in NSCLC tissues in comparison to normal tissues, and TIPRL could contribute to autophagy so as to facilitate NSCLC development through the eukaryotic initiation factor 2α (eIF2α)-Activating Transcription Factor 4 (ATF4) pathway [23]. In addition, TIPRL was found to correlated with lower overall survival rate of NSCLC patients; TIPRL knockdown suppressed NSCLC metastasis, indicating that TIPRL acted as an oncogene in NSCLC [36]. Similarly, our data also showed that TIPRL was upregulated in NSCLC. Moreover, exogenous introduction of TIPRL also attenuated hsa_circ_0010235 knockdown-induced NSCLC progression inhibition. Above results revealed the significant role of hsa_circ_0010235/miR-433-3p/TIPRL axis in NSCLC progression.

## Conclusion

In conclusion, hsa_circ_0010235 was upregulated in NSCLC. The tumor suppressor role of hsa_circ_0010235 knockdown in NSCLC progression in vitro and in vivo was testified. Our findings also corroborated that hsa_circ_0010235 affected NSCLC development by regulating miR-433-3p/TIPRL axis, highlighting a novel therapeutic target of NSCLC.

## Supplementary Information

**Additional file 1.** Raw images of Western blot results

## Data Availability

The analyzed data sets generated during the study are available from the corresponding author on reasonable request.
